# Protective immune responses against *Schistosoma mansoni* infection by immunization with functionally active gut-derived cysteine peptidases alone and in combination with glyceraldehyde 3-phosphate dehydrogenase

**DOI:** 10.1371/journal.pntd.0005443

**Published:** 2017-03-27

**Authors:** Hatem Tallima, Jan Dvořák, Sahira Kareem, Marwa Abou El Dahab, Nada Abdel Aziz, John Pius Dalton, Rashika El Ridi

**Affiliations:** 1 Zoology Department, Faculty of Science, Cairo University, Giza, Egypt; 2 Department of Chemistry, School of Science and Engineering, American University in Cairo, New Cairo, Cairo, Egypt; 3 School of Biological Sciences, Medical Biology Centre, Queen’s University Belfast, Northern Ireland, United Kingdom; 4 Zoology Department, Faculty of Science, Ein Shams University, Cairo, Egypt; 5 Chemistry Department, Faculty of Science, Cairo University, Giza, Egypt; Universidade Federal de Minas Gerais, BRAZIL

## Abstract

**Background:**

Schistosomiasis, a severe disease caused by parasites of the genus *Schistosoma*, is prevalent in 74 countries, affecting more than 250 million people, particularly children. We have previously shown that the *Schistosoma mansoni* gut-derived cysteine peptidase, cathepsin B1 (SmCB1), administered without adjuvant, elicits protection (>60%) against challenge infection of *S. mansoni* or *S*. *haematobium* in outbred, CD-1 mice. Here we compare the immunogenicity and protective potential of another gut-derived cysteine peptidase, *S*. *mansoni* cathepsin L3 (SmCL3), alone, and in combination with SmCB1. We also examined whether protective responses could be boosted by including a third non-peptidase schistosome secreted molecule, glyceraldehyde 3-phosphate dehydrogenase (SG3PDH), with the two peptidases.

**Methodology/Principal findings:**

While adjuvant-free SmCB1 and SmCL3 induced type 2 polarized responses in CD-1 outbred mice those elicited by SmCL3 were far weaker than those induced by SmCB1. Nevertheless, both cysteine peptidases evoked highly significant (*P* < 0.005) reduction in challenge worm burden (54–65%) as well as worm egg counts and viability. A combination of SmCL3 and SmCB1 did not induce significantly stronger immune responses or higher protection than that achieved using each peptidase alone. However, when the two peptidases were combined with SG3PDH the levels of protection against challenge *S*. *mansoni* infection reached 70–76% and were accompanied by highly significant (*P* < 0.005) decreases in worm egg counts and viability. Similarly, high levels of protection were achieved in hamsters immunized with the cysteine peptidase/SG3PDH-based vaccine.

**Conclusions/Significance:**

Gut-derived cysteine peptidases are highly protective against schistosome challenge infection when administered subcutaneously without adjuvant to outbred CD-1 mice and hamsters, and can also act to enhance the efficacy of other schistosome antigens, such as SG3PDH. This cysteine peptidase-based vaccine should now be advanced to experiments in non-human primates and, if shown promise, progressed to Phase 1 safety trials in humans.

## Author summary

Schistosomiasis, a severe disease caused by parasites of the genus *Schistosoma*, is prevalent in 74 countries in the Middle-east, Africa, South America and South-East Asia, and affects more than 250 million people, particularly children. Praziquantel is the most effective anti-schistosome drug and is the mainstay of mass drug administration (MDA) programs. However, concerns about the emergence of drug-resistant parasites or reduced efficacy have highlighted the urgent need for an efficacious vaccine that could interfere with parasite establishment and transmission. Here we examine the immunogenicity and vaccine potential of functionally active recombinant forms of the gut-derived cysteine peptidases of *S*. *mansoni*, cathepsin B1 (SmCB1) and cathepsin L3 (SmCL3), alone, or in combination. We demonstrate their capacity to induce high levels (>60%) of protection without the need of adjuvant against challenge infection with *S*. *mansoni* cercariae. A vaccine that combined SmCB1 and SmCL3 with another secreted molecule, *S*. *mansoni* glyceraldehyde 3-phosphate dehydrogenase (SG3PDH), induces predominant type 2 immune responses, and consistently evokes superior protection (>70%) against *S*. *mansoni* challenge infection in both outbred CD-1 mice and hamsters. This efficacious adjuvant-free cysteine peptidase-based vaccine should be brought forward into trials in non-human primates for assessment as a prospective vaccine against human schistosomiasis.

## Introduction

Schistosomiasis is caused by infection with helminth parasites of the genus *Schistosoma*. It is a water-borne debilitating disease that prevails in 74 developing countries of the Middle East, sub-Saharan Africa, and South America, and infects >250 million people. The infective stage, the cercaria, is shed by freshwater snails in large numbers and infects their human host by penetration of the skin. The parasites migrate via the blood to the lungs and then the liver before finally settling as male and female worms in the mesenteric (*Schistosoma mansoni*, *S*. *japonicum*) and bladder venules (*S*. *haematobium*). Schistosomiasis infects mainly rural populations, particularly children while they bath and play in freshwater tributaries [1]. Infection proceeds relatively unnoticed until eggs released by fecund females and destined to leave via the intestine or bladder, instead become trapped in the liver, gastrointestinal tract, or urinary bladder tissues. Here they induce potent inflammatory responses that lead to the development of severe granulomatous inflammation and fibrosis in the liver or bladder. In children, symptoms include anemia, abdominal pain and diarrhea, as well as growth and cognitive impairments [1–3].

Praziquantel is the only drug readily available for the treatment of schistosomiasis and because of its low cost, safety and efficacy has been the principal means of intervention to control schistosomiasis through mass drug administration (MDA) programs. Treatment with praziquantel, however, only reaches ~13% of the target population and because of the large pill size and bitterness it is generally not recommended for children below 6 years of age [4]. Furthermore, praziquantel does not prevent reinfection, therefore requiring repeated treatment, and has reduced efficacy in people with heavy infections [3,4]. There are fears surrounding the potential for the emergence of drug-resistant parasites [3] and a recent report from an MDA program in Uganda suggests that high exposure to the drug may reduce its effectiveness [5].

An efficacious and safe vaccine administered to young children would prevent infection and diminish transmission, as well as increase the likelihood of parasite elimination [1–3]. However, to date, only a few antigens have been advanced to Phase 1 trials in humans, including Sm-TSP-2 and Sm-14 for *S. mansoni* and Sh28GST (Bilhvax) for *S*. *haematobium*, while only one other, Smp80, is undergoing trials in non-human primates [3, 6–9]. The dearth of vaccines in the pipeline is worrying and therefore a major effort is required to employ new ways of discovering, shortlisting and delivering vaccine candidates.

Our research has focused on the identification of vaccine molecules in the parasite excretory-secretory products (ESP) because these are involved in host-parasite interaction and induce potent immune responses [10]. Contrary to cytosolic and surface membrane antigens, we reasoned that these were more accessible to attack by antibodies and activated immune effector cells [10]. We recently demonstrated that candidate vaccines in ESP formulated in the presence of the type 2 cytokines, thymic stromal lymphopoietin (TSLP), interleukin (IL)-25 or IL-33, induced considerably high protection in mice, in the range of 50% - 75%, against a challenge infection of *S*. *mansoni* cercariae [11]. We also discovered that a similar level of protection could be achieved by delivering antigens with the cysteine peptidase, papain, which also induces type 2 immune responses [11–13]. To avoid employing a plant-derived peptidase, we then showed that the gut-derived papain-like cysteine peptidases *of Schistosoma mansoni*, cathepsin B1 (SmCB1), and *Fasciola hepatica*, cathepsin L1 (FhCL1), could induce predominant type 2 immune responses and highly significant (*P* < 0.005) reduction of between 66% and 75% in challenge *S*. *mansoni* and *S*. *haematobium* worm burden and worm egg counts of outbred mice and hamsters [12, 14, 15].

The objectives of the present study were to compare and contrast the immunogenicity and protective potential of SmCB1 with another gut-derived schistosome cysteine peptidase, SmCL3 [16], alone and in combination in CD-1 outbred mice. We report that functionally active recombinant SmCB1 and SmCL3-based vaccine administered subcutaneously induces highly significant (*P* < 0.002) protection of > 60% against *S*. *mansoni* challenge infection in both mice and hamsters. A vaccine combining SmCB1 and SmCL3 with another secreted molecule, *S*. *mansoni* glyceraldehyde 3-phosphate dehydrogenase (SG3PDH), which is also located at the host-parasite interface [17–19], elicited impressive levels (>70%) of protection to *S*. *mansoni* challenge infection suggesting that this efficacious trivalent vaccine should now be brought forward into trials in non-human primates for assessment as a potential vaccine to control human schistosomiasis.

## Materials and methods

### Ethics statement

All animal experiments were performed following the recommendations of the current edition of the Guide for the Care and Use of Laboratory Animals, Institute of Laboratory Animal Resources, National Research Council, USA, and were approved by the Institutional Animal Care and Use Committee (IACUC) of the Faculty of Science, Cairo University, permit numbers CUFS F PHY 21 14 and CUFS-F-Imm-5-15.

### Parasites and animals

Female CD-1 mice and female Syrian hamsters (*Mesocricetus auratus*) were bred at the Schistosome Biological Materials Supply Program, Theodore Bilharz Research Institute (SBSP/TBRI), Giza, Egypt until 6 weeks of age and then maintained throughout experimentation at the animal facility of the Zoology Department, Faculty of Science, Cairo University. Cercariae of an Egyptian strain of *S*. *mansoni* were obtained from SBSP/TBRI, and used immediately after shedding from *Biomphalaria alexandrina* snails. CD1 mice were infected via whole body exposure as previously described [11, 13]. Hamsters were anesthetized, the abdomen shaved and wetted with sterile deionized water, and then exposed to cercariae via the ring method as previously described [12].

### Immunogens

Recombinant *S*. *mansoni* glyceraldehyde 3-phosphate dehydrogenase (rSG3PDH) expressed in the bacterium *Escherichia coli* was prepared and purified to homogeneity [17] (S1 Fig). This preparation contained <0.06 Endotoxin Units/ml as judged by the Pyrogen Gel-Clot Limulus Amebocyte Lysate test. Functionally active *S*. *mansoni* cathepsin B1 (SmCB1) and cathepsin L3 (SmCL3) were produced in methyltrophic yeast *Pichia pastoris* GS115 (Invitrogen) and PichiaPink (Thermo Fisher) strain, respectively, using methods previously described in our laboratory [20]. Recombinant cathepsins were purified by Ni-NTA column, desalted by dialysis, and stored in phosphate buffered saline, pH 7.3 at -80°C. Cathepsin peptidase purity was determined by sodium dodecyl sulfate-polyacrylamide gel electrophoresis (SDS-PAGE) and activity using the fluorogenic substrate Z-Phe-Arg-NHMec (Sigma-Aldrich, UK) and PolarStar Omega fluorescence reader (BMG Labtech) [20–22] (S1 Fig). Concentration of the purified peptidases was evaluated using the Protein Assay Kit of BioRad.

### Serum preparation

Blood samples were obtained from individual naïve, unimmunized and immunized mice (3 or 4 per group) seven days following infection with viable *S*. *mansoni* cercariae, unless stated otherwise. Sera were separated and stored at -20°C.

### Isolation and culture of epidermal, lymph node and spleen cells

Epidermal cells (EC) were isolated from three mice per group two days after *S*. *mansoni* infection, at the time when larvae are still resident in the epidermis, following the protocol described by Jensen et al. [23] with minor modifications. The whole procedure was recently detailed [13]. Lymph node cells (LNC) from inguinal and popliteal lymph nodes (LN) were recovered (3 mice per group) four days after *S*. *mansoni* infection at the time when larvae are in the dermis or dermal capillaries. LNC were also obtained from mice 21 days after immunization with vaccine molecules. Spleen cells (SC) were prepared from spleens removed from three mice per group at seven and 14 days after *S*. *mansoni* infection [11, 13, 14].

EC, LNC, and SC were re-suspended in RPMI-1640 medium supplemented with 200 U/ml penicillin, 200 μg/ml streptomycin, 25 mM HEPES, 50 ng/ml amphotericin, 5 x 10^–5^ M 2-mercaptoethanol, and 5% fetal calf serum (culture medium). Twenty μg/ml polymyxin B (Sigma-Aldrich) was added as an inhibitor of any residual lipopolysaccharide contamination of recombinant antigens. EC, LNC and SC were cultured at a concentration of 1 x 10^6^ cells/200 μl culture medium/well in duplicate wells of 96 round-bottomed well plates (Corning Costar, Bedford, MA), stimulated with 0 or 20 μg/ml membrane filter (0.45 μm)-sterilized immunogen, and maintained at 37°C in a humidified atmosphere containing 3.0% CO_2_. After 72 h of incubation, cultured cells were frozen and thawed for release of intracellular cytokines, the lysate centrifuged (15,000 x *g*) and supernatants stored at -76°C until assayed for cytokine concentrations by capture enzyme-linked immunosorbent assay (ELISA) [11, 13, 14].

### Immunological assays

Mouse serum antibody titer and isotype were assessed by indirect ELISA for binding to 250 ng/well immunogen in duplicate wells [11, 13, 14]. Horseradish peroxidase (HRP)-labeled goat anti-mouse IgG (H+L) conjugate (Promega, Madison, WI) was used to detect bound antibodies. Individual sera were serially diluted to determine the antibody titer and select the appropriate dilution for assessing the antibody isotypes. Based on these results, sera were diluted 1:100 or 1:200 to estimate the level of IgM and IgG class antibodies, and 1:25 for IgE and IgA antibodies. Biotin-labeled rat monoclonal antibody to mouse IgG1 (Pharmingen, San Diego, CA), IgA, and IgE (BioLegend, San Diego, CA), was diluted 1:500 in washing buffer (0.01 M phosphate buffered-saline, pH 7.2, 0.05% Tween 20/0.1% bovine serum albumin). Alkaline phosphatase- (AKP) or HRP-labeled streptavidin was diluted 1:1000. Monoclonal antibody to IgM, IgG2a and IgG2b (Pharmingen) labeled with AKP were diluted 1:500, 1:1000 and 1:1000, respectively. Reactivity was estimated spectrophotometrically after adding SureBlue TMB Microwell Peroxidase Substrate (Kirkegaard and Perry Laboratories, Inc., Gaithersburg, MD) or *p*-nitro phenyl phosphate (PNPP) substrate (Calbiochem, San Diego, CA).

Release of mouse IL-1β, IL-5, IL-12, IL-17, IL-25, IFN-γ, TSLP (ELISA MAX Set, BioLegend, San Diego, CA), and IL-13 (DuoSet ELISA Development System, R&D System Europe) was measured in supernatants of duplicate cell cultures by capture ELISA, following the manufacturer's instructions.

### Parasitological assays

Worm burden as well as liver and intestine worm egg load in individual mice and hamsters (5–10 animals per group) were evaluated six to seven weeks after the challenge infection with *S*. *mansoni* cercariae [11, 13, 15]. Percent (%) change was evaluated by the formula: % change = [mean number in unimmunized infected controls–mean number in immunized infected animals / mean number in infected controls] × 100.

Egg developmental stages were evaluated using 3–5 fragments of the distal portion of the ileum. After washing in 0.9% saline solution and slight drying on absorbent paper, each intestinal fragment was placed between 2 slides and analyzed by light microscopy to classify the eggs. For each fragment, up to 400 eggs were counted and classified according to their developmental stage as immature, with miracidium occupying less than two-thirds of the shell, mature containing an already developed miracidium, and non-viable, dead, clearly calcified, opaque [24].

### Experimental design of vaccine trials

**Fig 1:**
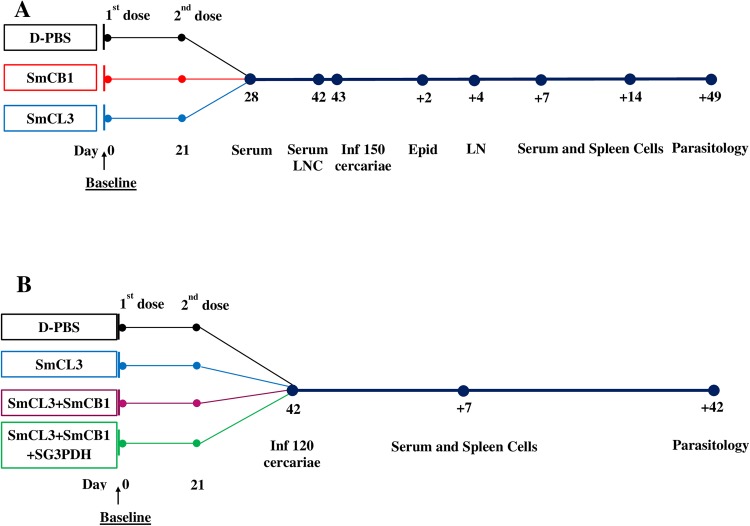
Overview of vaccine schedule using SmCB1 and SmCL3 in mice. Schedule shows regime of vaccine delivery and challenge infection with schistosome cercariae. Time-points of tissue sampling and parasitological analysis are also shown.

(a) Comparison of the immunogenicity and vaccine potential of SmCB1 and SmCL3 (Fig 1A). Seventy-five female CD-1 mice were divided into three groups of 25 mice each. Mice were injected subcutaneously twice, with a three week-interval, at the tail base region with 100 μl Dulbecco's phosphate-buffered saline, pH 7.1 (D-PBS) alone (control group) or containing 10 μg SmCB1 or SmCL3. Serum was obtained from three mice per group on days 7 and 21 after the second immunization. At 21 days, LNC were also assessed for cytokine release after three-day stimulation with the immunogen.

The remaining control and immunized mice were infected with 150 cercariae of *S*. *mansoni* 3 weeks after the second immunization. EC and LNC were isolated 2 and 4 days later, respectively from three mice/group, incubated with 0 or 20 μg/ml immunogen, and cell culture supernatants assessed for the levels of released TSLP, IL-1, IL-12, IL-25. On day 7 and 14, when a large proportion of migrating larvae are in the lung capillaries and liver sinusoids, respectively, serum and SC from three mice/group were assessed for humoral and cytokine responses to the immunogen. Parasitological parameters (see above) were evaluated for the remaining 6–8 mice per group at 49 days after the challenge infection (Fig 1A).

**Fig 2:**
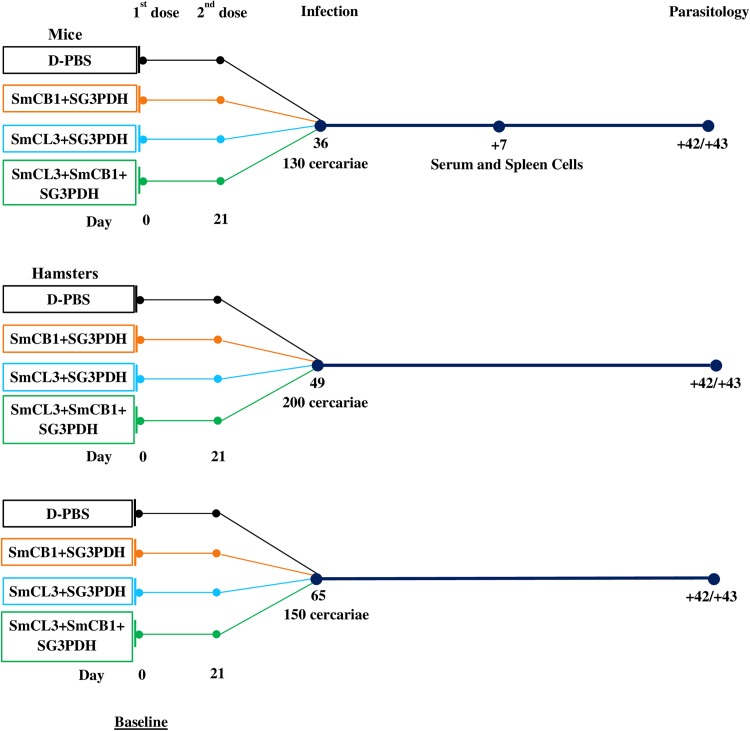
Overview of vaccine schedule using combination vaccine in mice and hamsters. Schedule show regime of vaccine delivery and challenge infection with schistosome cercariae. Time-points of tissue sampling and parasitological analysis are also shown.

(b) Evaluation of the immunogenicity and vaccine potential of SmCL3 alone or in combination with SmCB1 and SG3PDH (Fig 1B). Fifty-two CD-1 mice were randomly divided into 4 groups of 13 mice, and immunized subcutaneously twice, with a 3 week-interval, with 100 μl D-PBS containing 0 (control Group 1), 10 μg SmCL3 (Group 2), 10 μg SmCL3 and 10 μg SmCB1 administered subcutaneously on different sides of the tail (Group 3), 10 μg SmCL3 and 10 μg SmCB1 administered as above and 10 μg SG3PDH intramuscularly, to avoid immediate exposure to the cysteine peptidases (Group 4). Three weeks after the second immunization all mice were challenged with 120 cercariae of *S*. *mansoni*. Humoral and SC cytokine responses to the immunogens at seven days after infection were assessed using three mice per group. Parasitological parameters were evaluated for the remaining 10 mice per group at 42 days after the challenge infection (Fig 1B).

(c) Efficacy of a trivalent vaccine in mice and hamsters (Fig 2). Forty CD-1 mice were divided into groups of 10 mice, and immunized twice, with a three–week interval, with 0 (control group), 10 μg SmCB1 + 10 μg SG3PDH, 10 μg SmCL3 + 10 μg SG3PDH, and 10 μg SmCL3 + 10 μg SmCB1 + 10 μg SG3PDH (SG3PDH was given intramuscularly). All mice were challenged with 130 cercariae of *S*. *mansoni* two weeks after the last immunization. Humoral and SC cytokine responses to the immunogens at 7 days after infection were assessed using three mice per group. Parasitological parameters were examined for the remaining 7 mice on day 42 or 43 after challenge infection (Fig 2A).

To assess the vaccine in a different host, 40 hamsters were allocated into 4 groups of 10 animals, and immunized following a protocol similar to that described in (c) above with the exception that these were administered 15 μg rather than 10 μg of each immunogen for both immunizations. Five hamsters in each group were challenged 4 and 6 weeks after the last immunization with 200 or 150 cercariae (Fig 2B), respectively. Parasitological parameters were examined for all hamsters on day 42 or 43 after challenge infection.

### Statistical analyses

All values were tested for normality. Mann–Whitney 2-tailed test was used to analyze the statistical significance of differences between experimental and control values and considered significant at *P* < 0.05.

## Results

### Immunogenicity and protective capacity of *S*. *mansoni* cysteine proteases cathepsin B1 (SmCB1) and cathepsin L3 (SmCL3)

**Fig 3:**
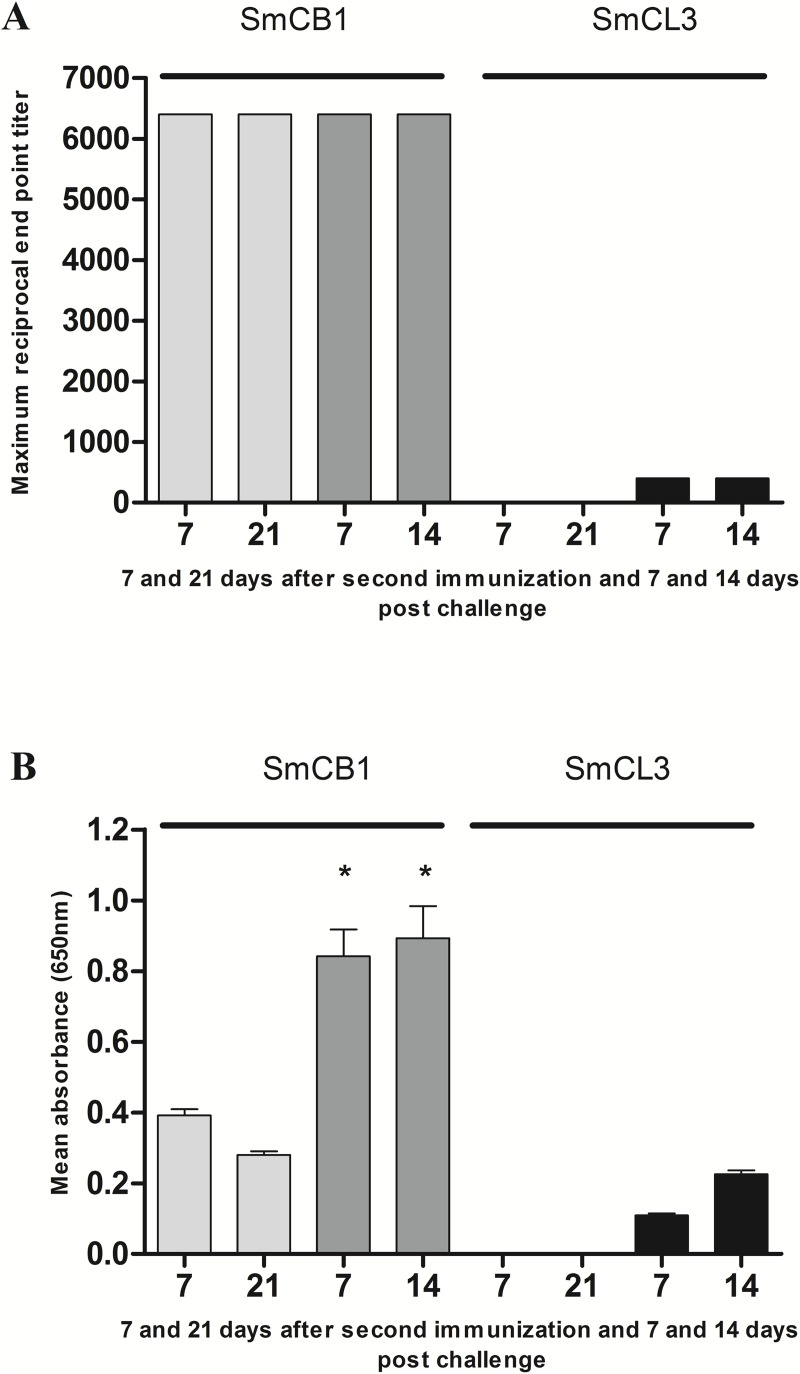
Serum antibody response to SmCB1 and SmCL3 following immunization and challenge infection of mice. (A) Serum antibody reactivity to SmCB1 and SmCL3 was assessed by ELISA at 7 and 21 days after second immunization and 7 and 14 days following challenge infection with 150 cercariae of *S*. *mansoni*. (B) Antibody reactivity at 7 and 21 days after second immunization and 7 and 14 days after the challenge infection were compared at dilutions of 1:200 with each column denoting mean value for three mice +/- SD; asterisks indicate significance (* *P* < 0.05) of differences.

We have previously demonstrated the ability of two immunizations with functionally active SmCB1 to protect mice against a challenge infection of *S*. *mansoni*. Here we have compared a second cysteine protease, SmCL3 that is also expressed in the *S*. *mansoni* digestive tract [16], to SmCB1 for its capacity to induce immune responses and protection against parasite challenge. In keeping with previous results [14] subcutaneous immunization of mice with SmCB1 induced IgG antibody responses (titres >1:6400) which can be detected at 7 and 21 days after the second immunization (Fig 3, panel A). At 7 and 14 days following the challenge infection with 150 cercariae the antibody responses to SmCB1 were significantly (*P* < 0.05) boosted as shown by ELISA performed at serum dilutions of 1:200 (Fig 3, panel B). Antibodies binding to SmCB1 were of the IgM, IgG1, and IgG2b isotype to a titre of 1:400 whereas little or no IgE was detected (titre of 1:25). By contrast, we did not detect any antibodies to SmCL3 at 7 or 21 days after two immunizations. Although, antibodies to SmCL3 were detected following parasite challenge, these were of very low titre and markedly lower than those elicited by SmCB1 (Fig 3, panel B).

**Fig 4:**
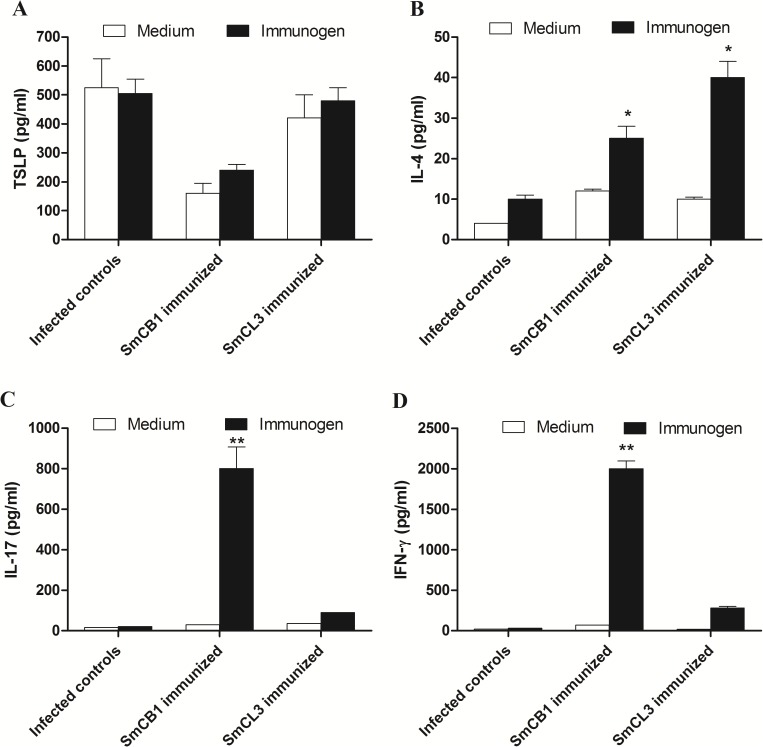
Cytokine response of SmCB1- and SmCL3- immunized mice to the immunogen. Each column represents mean +/- SD cytokine levels in epidermal (A) and lymph node (B-D) cell cultures (three mice per group), 2 (epidermal cells, A) and 4 (LNC, B-D) days after infection. Asterisks indicate significance (* *P* < 0.05, ** *P* < 0.005) of differences between levels of cytokines released in cultures stimulated with 0 (medium) or 20 μg/ml immunogen.

To examine early immune responses in the skin of immunized and challenged mice, EC were removed two days after the challenge infection, incubated in the presence or absence of SmCB1 and SmCL3 and secretion of the cytokine measured. Epidermal cells from control or SmCB1- or SmCL3-immunized mice did not release detectable levels of IL-1, IL-12, or IL-25 following stimulation in culture for 72 h with 0 or 20 μg/ml immunogen. However, both unstimulated and immunogen-stimulated EC from the test mice did release detectable levels of TSLP with levels produced by EC of SmCB1-immunized mice significantly (*P* < 0.05) lower compared to infected controls and SmCL3-immunized mice (Fig 4A).

Inguinal and popliteal LNC taken from mice 4 days after infection released low but significant (*P* < 0.05) levels of IL-4 in response to stimulation with SmCB1 or SmCL3 (Fig 4B) but only SmCB1 induced significant (*P* < 0.005) levels of IL-17 (Fig 4C) and IFN-γ (Fig 4D). Similar cytokine responses were observed for SC taken at 7 and 14 days after the challenge infection and stimulated with SmCB1 and SmCL3 (S2 Fig).

**Fig 5:**
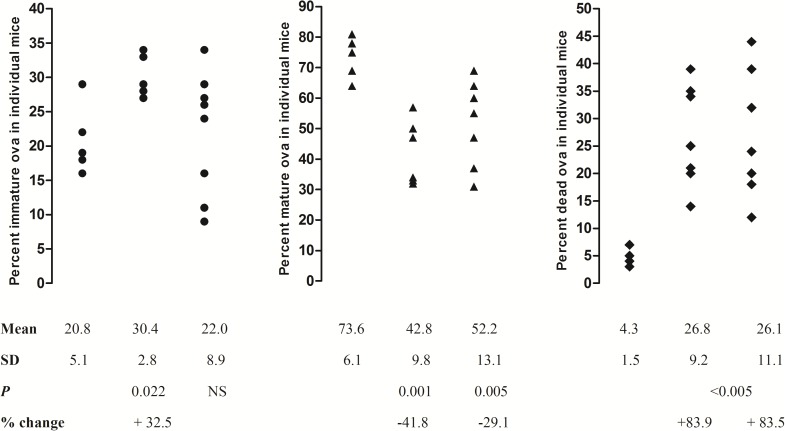
Effect of immunization with SmCB1 and SmCL3 on *S. mansoni* egg development. Percent immature (left box), mature (middle box) and dead (right box) ova was assessed seven weeks after infection with 150 *S*. *mansoni* cercariae in CD1 mice (6-8/group) that were unimmunized (left column in each box) or immunized with SmCB1 (middle column in each box) or SmCL3 (right column in each box). *P* values as assessed by the Mann-Whitney test; NS = not significant.

Both SmCB1 and SmCL3 vaccine antigens induced highly significant (*P* < 0.005) reduction in total worm burdens (54.2% and 65.4%, respectively) that was reflected in comparable reduction in both male and female worms together with a significant (*P* < 0.05) decrease in worm egg counts in the liver and small intestine tissues of mice (Table 1). No significant differences were observed between SmCB1 and SmCL3 vaccine efficacy and both immunizations also induced highly significant (*P* < 0.005) increase in the percentage of dead ova compared to the infected control mice (Fig 5).

**Table 1:** Effect of immunization with SmCB1 or SmCL3 on parasitological parameters of challenge *S*. *mansoni* infection in outbred mice seven weeks post infection.

	Infected Controls	SmCB1 immunized	SmCL3 immunized
**Total worm burden**			
Mean ±SD	99.0 ± 10.7	40.8 ± 10.8	34.2 ± 4.6
*P* value		0.0016	0.0025
Reduction (%)		58.7	65.4
**Male worm burden**			
Mean ±SD	56.4 ± 8.6	21.3 ± 6.2	18.2 ± 2.9
*P* value		0.0016	0.0025
Reduction (%)		62.2	67.7
**Female worm burden**			
Mean ±SD	42.6 ± 4.5	19.5 ± 4.8	16.0 ± 2.1
*P* value		0.0016	0.0012
Reduction (%)		54.2	62.4
**Liver egg counts**			
Mean ±SD	46166 ± 14986	27142 ± 10205	33000 ± 828
*P* value		0.035	0.049
Reduction (%)		41.2	28.5
**Intestine egg counts**			
Mean ±SD	60833 ± 21646	38142 ± 13993	43428 ± 7325
*P* value		0.048	0.032
Reduction (%)		37.3	28.6

Mice (6–7 per group) were challenged 3 weeks after second immunization with 150 cercariae of *S*. *mansoni* and assessed for parasitological parameters seven weeks post infection. NS = not significant, as assessed by the Mann-Whitney test (two-tailed *P* value). Reduction % = mean number in unimmunized mice–mean number in cysteine peptidase- immunized mice/ mean number in unimmunized mice x 100.

### Immunogenicity and protective capacity of *S*. *mansoni* cysteine protease SmCL3 alone and in combination with SmCB1 and SG3PDH

**Fig 6:**
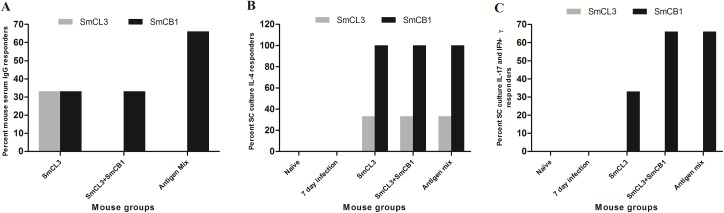
Immunogenicity of SmCL3 alone and in combination with SmCB1 and SG3PDH. (A) Percent mice with serum IgG antibody binding to SmCL3 or SmCB1 significantly (*P* < 0.05) higher than naïve and 7-day infection mice is represented by grey and black columns, respectively (B & C). *Ex vivo* spleen cells from 3 mice from each group of naïve, 7-day infection, SmCL3-, SmCL3 + SmCB1-, or SmCL3+SmCB1+ SG3PDH (Antigen Mix)-immunized mice were stimulated 7 days after infection with *S*. *mansoni* with 20 μg/ml SmCL3 (grey columns) or SmCB1 (black columns) and spleen supernatants assayed for release of cytokines by capture ELISA. Each column represents percent mouse SC culture releasing cytokine detectable levels, higher than mean of naïve and controls + 2 SD.

Consistent with the above experiments we found that only one out of 3 mice immunized with SmCL3 produced antibodies that bound to SmCL3 in levels significantly (*P* < 0.05) higher than unimmunized 7 day infected mice. Addition of SmCB1 and SG3PDH to SmCL3 did not influence production of anti-SmCL3 antibodies even though antibodies to SmCB1 (to a titre higher than 1:800) were induced in mice immunized with SmCL3 + SmCB1 (Fig 6A). No SmCL3- or SmCB1-binding IgE antibodies were detected even at 1:25-diluted serum from naïve or immunized mice from all groups, confirming the data revealing that SmCL3 vaccine does not induce IgE specific antibodies, and that SmCB1 is a weak IgE specific antibody inducer, but not when incorporated with SG3PDH [14].

SC taken from non-infected or unimmunized control CD-1 mice that were infected for 7 days with *S*. *mansoni* did not release detectable levels of IL-4, IL-5, IL-13, IL-17 or IFN-γ when stimulated with SmCL3 or SmCB1. SC obtained 7 days after infection with *S*. *mansoni* from mice that were immunized with SmCL3 alone, SmCL3 + SmCB1 or SmCL3+ SmCB1 + SG3PDH did not release IL-5, IL-13, IL-17 or IFN-γ when stimulated with SmCL3, yet released IL-4 in response to SmCL3 and especially SmCB1, similarly to the observation reported previously with SmCB1 and FhCL1 [14]. The SC response to SmCB1 was strikingly different from SmCL3 as the former induced greater percent of mice responding with detectable levels of IL-17 or IFN-γ (Fig 6B and 6C).

**Fig 7:**
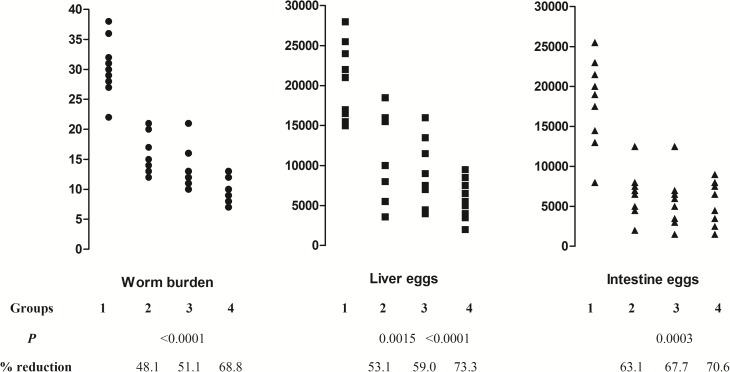
Effect of immunization with SmCL3 alone or with SmCB and SG3PDH on parasitological parameters of challenge *S*. *mansoni* infection in outbred mice. Mice (10 per group) unimmunized (Group 1), immunized with SmCL3 (Group 2), SmCL3 + SmCB1 (Group 3) or SmCL3 + SmCB1+ SG3PDH (Group 4) were challenged 3 weeks after second immunization with 120 cercariae of *S*. *mansoni* and assessed for parasitological parameters 6 weeks post infection. *P* values as assessed by the Mann-Whitney test (two-tailed). Reduction % = mean number in unimmunized mice–mean number in immunized mice/ mean number in unimmunized mice x 100.

Consistent with the first vaccine experiment reported above, CD-1mice immunized twice subcutaneously with functionally active SmCL3 protease displayed a highly significant (*P* < 0.0001, Mann-Whitney) reduction in challenge worm burdens (48%) compared to the control mice when challenged with *S*. *mansoni* cercariae three weeks after the second immunization. The level of protection was not significantly increased when SmCL3 and SmCB1 were employed as a cocktail vaccine (reduction of 51.1% in worm burdens). However, significantly (*P* = 0.0035) higher protection of 69% was observed when the vaccine was composed of SmCL3 + SmCB1 + SG3PDH (Fig 7A). Furthermore, immunization of CD-1 mice with SmCL3 + SmCB1 was associated with highly significant decrease in parasite egg load in liver and small intestine (53% and 59%, respectively, *P* <0.0001) which was further decreased when SG3PDH was added to the vaccine cocktail (73% and 70%, respectively, *P* <0.0001) (Fig 7B and 7C).

### Comparative efficacy of vaccines in outbred mice and hamsters

**Fig 8:**
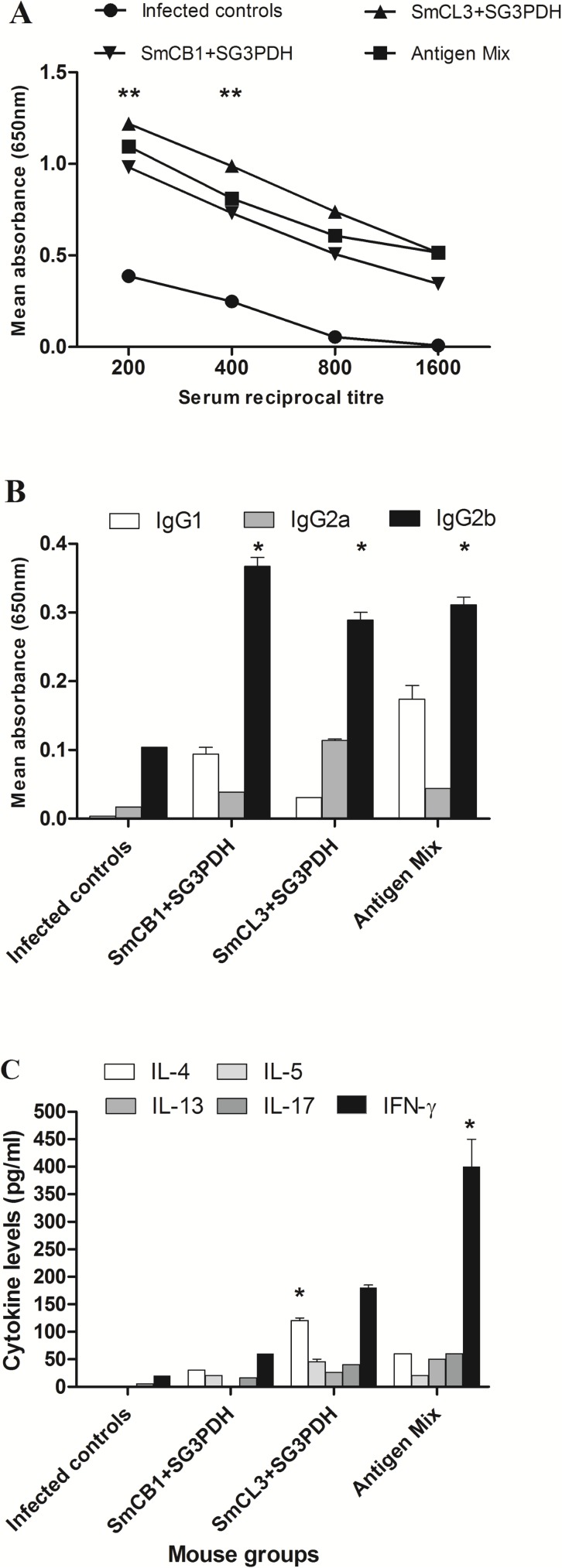
Mouse serum antibody and cytokine response to SG3PDH delivered with cysteine peptidases. Sera taken from control and immunized [SmCB1+ SG3PDH, SmCL3+ SG3PDH and SmCB1+SmCL3+ SG3PDH (Antigen Mix)] mice obtained 7 days after challenge *S*. *mansoni* infection were analyzed for antibody titers (A) and isotype (B) to SG3PDH in ELISA. Spleen cells obtained at the same time point were analyzed for cytokine responses following in vitro stimulation with 0 or 20 μg/ml SG3PDH (C). Each point or column represents mean values +/- SD for three mice per group. Asterisks indicate significance (* *P* < 0.05, ** *P* < 0.005) of differences between immunized & infected and control-infected mice.

A third series of vaccines trials was performed to support the protective efficacy of SmCB1 and SmCL3 and to compare this data in two laboratory outbred models, CD-1 mice and hamsters. The focus of the previous experiments was to compare the immunogenicity of SmCB1 and SmCL3. The focus in the third experiment was to compare the adjuvant effects of SmCB1 and SmCL3 on responses to SG3PDH. Accordingly, we analyzed the antibody response to SG3PDH in the presence of the peptidase in the vaccine cocktail (Fig 8A and 8B). Antibody reactivity to SG3PDH was observed in challenged mice immunized with SmCB1+ SG3PDH, SmCL3+ SG3PDH or SmCB1+SmCL3+ SG3PDH and was significantly (*P* < 0.005) higher than non-immunized mice (Fig 8A). The adjuvant effect of SmCB1 and SmCB1 + SmCL3 on mouse humoral reactivity to SG3PDH was characterized by the production of substantial amounts of IgG1 antibodies (Fig 8B) which contrasts with our previous studies showing that murine humoral responses to SG3PDH administered alone is limited to IgG2a and IgG2b [10, 11]. Cytokine production by SC in response to SG3PDH was characterized by the detection of low but significant levels of IL-4, IL-5, IL-13, IL-17 and particularly IFN-γ (Fig 8C).

The combination of SmCB1 or SmCL3 with SG3PDH as a vaccine cocktail induced highly significant (*P* <0.02 - <0.008) reduction in challenge worm burden in immunized CD-1 mice (66–71%) and hamsters (51–66%) compared to unimmunized animals. A very significant (*P* = 0.0006) reduction of 76% was achieved in total worm burden (reflected in both male and female challenge worms) in mice given the vaccine formula SmCB1 + SmCL3 + SG3PDH. The cocktail vaccine also elicited highly significant (*P* < 0.008) decrease (66% - 74%) in challenge worm burden in hamsters, challenged either 4 or 6 weeks after the second immunization (Table 2). The decrease in total egg counts in liver and intestine was less pronounced in hamsters compared to mice. However, SmCB1 or SmCL3 + SG3PDH and SmCB1 + SmCL3 + SG3PDH did induce significant (*P* < 0.05) reduction in percentage of immature ova and highly significant (around *P* = 0.0001) increase of >80% in the percentage of dead ova in small intestine of immunized as compared to unimmunized hosts (Table 2).

**Table 2:** Effects of the cysteine peptidase-based vaccine on challenge worm parasitological parameters six weeks post infection.

	% Reduction in total*					
Vaccine formulation	Worms	Eggs (liver)	Eggs (intestine)	% Reduction in immature ova	% Reduction in mature ova	% Increase in dead ova**
**1. CD1 mice challenged 2 weeks after last immunization with 120 cercariae**						
SmCB1 + SG3PDH	66.1 (0.0012)	43.4 (0.014)	NS	33.1 (0.035)	NS	73.2 (0.0001)
SmCL3 + SG3PDH	70.9 (0.0012)	56.7 (0.0012)	42.8 (0.035)	70.6 (0.0002)	NS	81.9 (0.0004)
SmCB1 + SmCL3 + SG3PDH	76.5 (0.0006)	61.6 (0.0006)	57.1 (0.0023)	58.7 (0.0003)	NS	87.4 (0.0001)
**2. Hamsters challenged 4 weeks after last immunization with 200 cercariae**						
SmCB1 + SG3PDH	58.1 (0.016)	NS	NS	28.9 (0.049)	NS	81.2 (0.0001)
SmCL3 + SG3PDH	53.4 (0.0079)	NS	34.5 (0.028)	44.6 (0.011)	NS	80.5 (0.0001)
SmCB1 + SmCL3 + SG3PDH	66.2 (0.0079)	30.1 (0.016)	32.6 (0.028)	27.1 (0.048)	38.7 (0.045)	82.3 (0.0001)
**3. Hamsters challenged 6 weeks after last immunization with 150 cercariae**						
SmCB1 + SG3PDH	50.8 (0.0079)	NS	NS	NS	NS	80.2 (0.0077)
SmCL3 + SG3PDH	62.5 (0.0079)	25.5 (0.048)	28.8 (0.043)	42.8 (0.021)	NS	83.1 (0.0004)
SmCB1 + SmCL3 + SG3PDH	74.1 (0.005)	41.3 (0.013)	32.9 (0.027)	38.5 (0.038)	NS	81.5 (0.0001)

Mice (7 per group) and hamsters (5 per group) were evaluated for parasitological parameters six weeks post infection with cercariae of *S*. *mansoni*.

*Percent reduction was evaluated by the formula: % reduction = mean number in infected controls − mean number in infected, treated hosts/mean number in infected controls × 100. *P* values as calculated by 2-tailed Mann-Whitney test, in brackets.

**Percent increase was evaluated by the formula: % increase = − mean number in infected, treated hosts − mean number in infected controls/mean number in infected, treated hosts × 100. NS = not significant.

## Discussion

There is an urgent need to develop vaccines for schistosomiasis given our almost complete dependency on the drug praziquantel and the lack of new compounds entering the development pipeline [4]. Peptidases, particularly those involved in the degradation of blood proteins for nutrient, have for a long time been considered important targets at which we could direct both drugs and vaccines [21, 22]. Cathepsin B, SmCB1, is one of the most prominent and immunogenic peptidase secreted from the gastrodermis of schistosomes [20–22, 25]. Recently we discovered that this enzyme could induce high levels of protection in mice against a challenge infection with cercariae of *S*. *mansoni* when delivered subcutaneously as a functionally active recombinant form without adjuvant [14, 15]. The protection afforded by active cathepsin B was suggested to be partly due to its ability to activate components of the innate immune system in a non-specific manner as the cysteine peptidase papain from plants [11, 13] and the papain-like cathepsin L1, FhCL1, of the related trematode *Fasciola hepatica* [14, 15], also had the capacity to induce protection against *S*. *mansoni*. Notwithstanding, we felt it judicious to pursue SmCB1 as a protective vaccine molecule rather than these other peptidases for schistosomiasis because it was derived from the parasite itself. Furthermore, we found that inactive SmCB1 also possesses protective properties, albeit lower than active cathepsin B [14], and recently Ricciardi et al. [26] showed that SmCB1 formulated in Montanide ISA 720 VG induced elicited high-level protection against *S*. *mansoni*. In the present study, we pursued another schistosome peptidase that was recently shown to be located in the schistosome digestive tract, *S*. *mansoni* cathepsin L3 (SmCL3) [16], and investigated its immunogenicity and protective properties alone and in combination with the cathepsin B peptidases without adjuvant. To do this we produced a functionally active SmCL3 protease in the methylotrophic yeast *Pichia pastoris* similar to the manner in which we produced SmCB1 [20].

To our surprise, we found that SmCL3 induced weaker humoral immune responses when administered subcutaneously to outbred CD-1 mice compared to SmCB1 despite that SmCL3 displays only 46% identity with mouse cathepsin L [16]. Two immunizations of SmCL3 elicited no detectable IgG antibody responses to the immunogen at 7 and 21 days after secondary immunization, reflecting an inability to induce T helper cells and release of cytokines that promote antibody production. The SmCL3 poor immunogenicity could be ascribed to its uniquely broad proteolytic substrate specificity [16]. In support, the inability of SmCL3 to strongly activate the cellular arm of the host immune system was also observed *in vitro* whereby *ex vivo*-stimulated SC released low, albeit detectable, levels of IL-4. By contrast, SmCB1 induced a greater level of cytokine responses from LNC and SC and these were not limited to the type 2 cytokine IL-4 as IL-17 and IFN-γ were also detected. Furthermore, IgM, IgG1, IgG2a and IgG2b antibodies to SmCB1 were produced *in vivo* at 7 and 21 days after secondary immunization. Although a low level of anti-SmCL3 antibody was detected following a challenge infection of vaccinated mice these were far lower than those observed for SmCB1, suggesting a more prominent infection-associated anamnestic response for the latter peptidase which is supported by it well-known immunogenicity in experimental and natural infection [27]. However, both peptidases stimulated detectable levels of TSLP from EC cells *in vitro* that suggests a role for this master cytokine of type 2 immune responses [28, 29] in initiating protective responses. Our previous studies have shown that delivery of TSLP to mice induced type-2 related protective immune responses against *S*. *mansoni* challenge infection [11], which could have involved the recruitment and activation of eosinophils, basophils, and mast cells [29–36].

Despite the lower immune responses induced by SmCL3 compared to SmCB1, the peptidase elicited a highly significant (*P* < 0.005) reduction (~65%) in challenge *S*. *mansoni* worm burden, and not significantly different from the protection levels induced by SmCB1 (59%). In this trial immunization with either *S*. *mansoni* cysteine peptidase was associated with low but significant (*P* < 0.05) decreases in parasite eggs within the intestine and liver in comparison to the infected controls, and a very significant effect (*P* < 0.005) on the maturation and viability of eggs in these tissues observed. This is relevant as dead ova are likely unable to elicit inflammatory responses in the host or participate in transmission of the infection [24]. The data may indicate that cysteine peptidase-induced immune responses that may not only act directly on the female worm reproductive system via reducing fecundity but also interfere somehow in the development of the embryo within the egg. Perhaps the latter effect is due a blocking of the ability of female worms to digest blood proteins effectively and, consequently, acquire sufficient amino acids for egg protein synthesis.

In repeat experiments, an analysis of immune responses to SmCL3 delivered in combination with SmCB1 or SG3PDH, confirmed its lack of ability to elicit production of considerable levels of SmCL3-specific antibodies (IgG1 antibodies were observed but only to a titer of 1:25) and to stimulate SC *ex vivo* barely detectable levels of IL-4. Despite this apparent lack of immunogenicity significant (48%, *P* < 0.0005) reduction in total worm burden and parasite egg load in liver and small intestine at three weeks after infection was observed. However, the protective potential of SmCL3 was not increased upon inclusion of SmCB1 to the vaccine. Likewise, we previously found that a combination of FhCL1 and SmCB1 peptidases did not elicit the additive protective effect of each peptidase given alone [14]. However, inclusion of SG3PDH with the SmCB1 and SmCL3 cysteine peptidases increased protection to >69% in *S*. *mansoni* worm burden and improved on the vaccine’s capacity to reduce egg loads in the liver and small intestine tissues. The enhanced protection could be associated with immune responses directed against lung-stage larvae that express SG3PDH on their surface and also release it as a component of their ESP [18, 19].

In a final series of vaccine trials (shown in Table 2) we set out to support the data obtained in previous tests but also to compare the protective potential in mice and hamsters. Furthermore, in the case of hamsters, the time period between the second immunization and challenge infection with *S*. *mansoni* cercariae was compared at four and six weeks. These studies were also motivated by the recently published discussion article by Wilson and colleagues [37] who argued that the mouse model may give spurious vaccine results due to physiological features of the murine pulmonary system (non-specific, inflammation-associated leakiness of the lung vasculature that drive parasites into the alveoli) [37]. Our previous vaccine trials with cysteine peptidases [11, 14, 15] were cited as an example, if not evidence, of a vaccine that could induce these non-specific responses. In these trials in mice, antibody and cytokine responses to SG3PDH were observed and revealed that, (a) IgG responses to SG3PDH was comparable in all three groups combination vaccines, SmCL3 + SG3PDH, SmCB1 + SG3PDH and SmCB1 + SmCL3 + SG3PDH and comprised IgG1 and IgG2 isotypes, and (b) type 1, 2 and 17 cytokine were detected in all groups indicating the induction of mixed Th1/Th2 responses, although the group given SmCB1 + SmCL3 + SG3PDH exhibited a more skewed Th1/Th17 response to SG3PDH (Fig 7). These responses are similar to the profile observed when SG3PDH was co-administered with the cytokines TSLP, IL-25 and IL-33 [11]. The protection reported by Ricciardi et al. [26] using Montanide-formulated SmCB1 also was attributed to a mixed Th1/Th2/Th17 response. Most importantly, the data revealed that combining SmCB1 or SmCL3 with SG3PDH provided an adjuvant-like effect that was accompanied by highly significant (*P* < 0.05- <0.005) reduction in challenge worm and worm egg burden and the proportion of immature ova (Table 2), Consistent with the observed potent antibody and cellular immune responses to SG3PDH, the protection level achieved with the complete vaccine formula, SmCB1 + SmCL3 + SG3PDH, was remarkably high, ~76% in total worm burden. High levels of protection were also observed in vaccinated hamsters with SmCB1 or SmCL1 and SG3PDH (50–62%), with particularly high reductions of 66% and 74.1% in total worm burden recorded in hamsters challenged four and six weeks after the second immunization with SmCB1 + SmCL3 + SG3PDH. These protective anti-worm data were accompanied with consistent, reproducible, and highly significant increases in the number of dead ova within the small intestine.

It has been several decades since the World Health Organization (WHO) proposed a threshold level of 40% reduction in challenge worm burden in an experimental host for sponsoring a schistosome vaccine for pre-clinical trials and still very few molecular vaccines have passed this relatively-low bar [3, 37]. Here we provide further evidence for the protective capacity of cysteine peptidases when administered subcutaneously in functionally active form. The mechanism by which cysteine peptidase elicit protective responses is still not fully understood, especially considering their low immunogenicity when administered in this fashion (particularly SmCL3, as observed in this study), but we suggest that a contributing factor is the provocation of a Th2 or Th1/Th2/Th17 immune response by activating innate immune responses. Further work is required to explore whether the mechanism activation of immune responses by cysteine proteases and whether this can be enhanced by other protective surface or ESP antigens in order to promote greater anti-parasite activity as we have shown in this study with SG3PDH. Although we have not addressed all the suggestions of Wilson et al. [37] to test their assertions that the mouse model is one that predisposes towards spurious or misleading vaccine results, we have at least demonstrated that protection can be induced in another rodent model hamster, and that protection levels are retained even when he challenge infection is given six weeks after the final immunization. It must be also emphasized that our vaccine formulation is delivered without adjuvant and, therefore, the likelihood of non-specific physiological factors being involved in protection in lessened. We believe that the multiple trial data in the present study justifies the candidacy of the cysteine peptidase-based vaccine for funding to support independent trials in mice and non-human primates and, if shown promise, should be positioned in a development pipeline for human Phase 1 trials.

## Supporting information

S1 Fig SmCB1 (A) and SmCL3 (B) recombinant proteins were produced in Queen’s University Belfast using the yeast *Pichia pastoris* as the expression system. The double band of SmCB1 and the low band of SmCL3 are evidence of proteolytic processing of the zymogen enzymes to mature form during the isolation procedure. (C) The proteases were demonstrated to be functionally active with SmCB1 and SmCL3 having a specificity activity of 30,000 and 60,000 rfu/μg over 4 mins at 37°C against the fluorogenic peptide substrate Z-Phe-Ala-NHMec (see reference 20). (D) Purified rSG3PDH, prepared at Cairo University, migrated at 40 kDa. Enzymatic analysis determined that the purified recombinant protein possesses distinct GAPDH activity as assessed by the Glyceraldehyde 3 Phosphate Dehydrogenase Activity Colorimetric Assay Kit (ab204732, Abcam, Cambridge, MA). The specific activity was approximately 30% of that observed for control rabbit GAPDH.
(PPTX)

S2 Fig Typical cytokine response of SmCB1- and SmCL3- immunized mice to the immunogen 7 or 14 days after challenge infection. Each column represents mean cytokine levels +/- SD released by spleen cells of three mice per group 14 days after infection. Asterisks indicate significance (* *P* < 0.05, ** P < 0.005) of differences between levels of cytokines released in cultures stimulated with 0 (medium) or 20 μg/ml immunogen.
(PPTX)
