# Supplementary material for: Protective immune responses against Schistosoma mansoni infection by immunization with functionally active gut-derived cysteine peptidases alone and in combination with glyceraldehyde 3-phosphate dehydrogenase
Source: PLoS Negl Trop Dis. 2017 Mar 27;11(3):e0005443. doi: 10.1371/journal.pntd.0005443 (PMC5386297; doi:10.1371/journal.pntd.0005443)
Supplement: S1 Fig — SmCB1 (A) and SmCL3 (B) recombinant proteins were produced in Queen’s University Belfast using the yeast Pichia pastoris as the expression system. The double band of SmCB1 and the low band of SmCL3 are evidence of proteolytic processing of the zymogen enzymes to mature form during the isolation procedure. (C) The proteases were demonstrated to be functionally active with SmCB1 and SmCL3 having a specificity activity of 30,000 and 60,000 rfu/μg over 4 mins at 37°C against the fluorogenic peptide substrate Z-Phe-Ala-NHMec (see reference 20). (D) Purified rSG3PDH, prepared at Cairo University, migrated at 40 kDa. Enzymatic analysis determined that the purified recombinant protein possesses distinct GAPDH activity as assessed by the Glyceraldehyde 3 Phosphate Dehydrogenase Activity Colorimetric Assay Kit (ab204732, Abcam, Cambridge, MA). The specific activity was approximately 30% of that observed for control rabbit GAPDH. (PPTX) [file pntd.0005443.s001.pptx]

## Slide 1
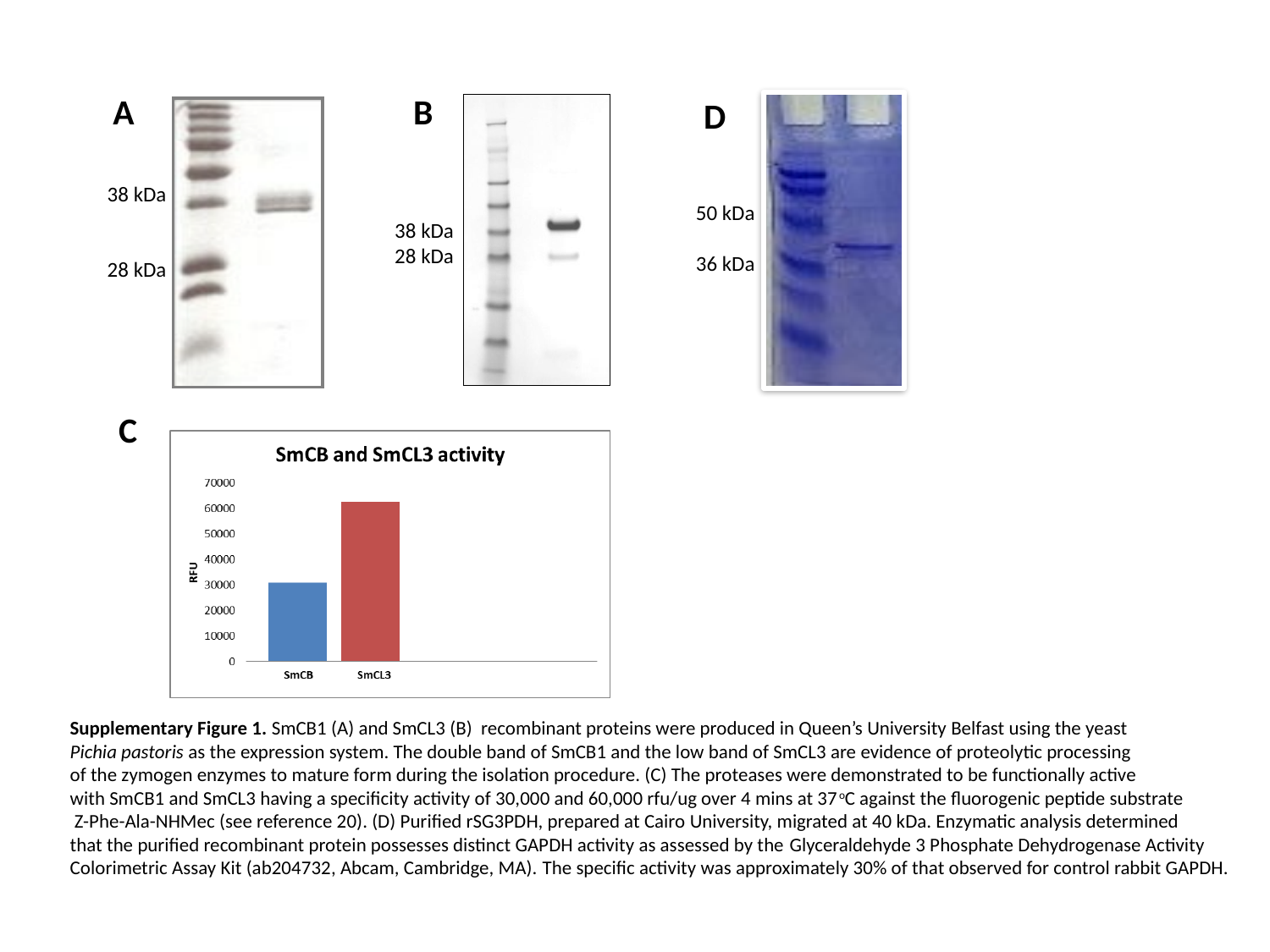

A
B
D
38 kDa
28 kDa
50 kDa
36 kDa
38 kDa
28 kDa
C
Supplementary Figure 1. SmCB1 (A) and SmCL3 (B) recombinant proteins were produced in Queen’s University Belfast using the yeast
Pichia pastoris as the expression system. The double band of SmCB1 and the low band of SmCL3 are evidence of proteolytic processing
of the zymogen enzymes to mature form during the isolation procedure. (C) The proteases were demonstrated to be functionally active
with SmCB1 and SmCL3 having a specificity activity of 30,000 and 60,000 rfu/ug over 4 mins at 37oC against the fluorogenic peptide substrate
 Z-Phe-Ala-NHMec (see reference 20). (D) Purified rSG3PDH, prepared at Cairo University, migrated at 40 kDa. Enzymatic analysis determined
that the purified recombinant protein possesses distinct GAPDH activity as assessed by the Glyceraldehyde 3 Phosphate Dehydrogenase Activity
Colorimetric Assay Kit (ab204732, Abcam, Cambridge, MA). The specific activity was approximately 30% of that observed for control rabbit GAPDH.
