# Supplementary material for: Protective immune responses against Schistosoma mansoni infection by immunization with functionally active gut-derived cysteine peptidases alone and in combination with glyceraldehyde 3-phosphate dehydrogenase
Source: PLoS Negl Trop Dis. 2017 Mar 27;11(3):e0005443. doi: 10.1371/journal.pntd.0005443 (PMC5386297; doi:10.1371/journal.pntd.0005443)
Supplement: S2 Fig — Each column represents mean cytokine levels +/- SD released by spleen cells of three mice per group 14 days after infection. Asterisks indicate significance (* P < 0.05, ** P < 0.005) of differences between levels of cytokines released in cultures stimulated with 0 (medium) or 20 μg/ml immunogen. (PPTX) [file pntd.0005443.s002.pptx]

## Slide 1
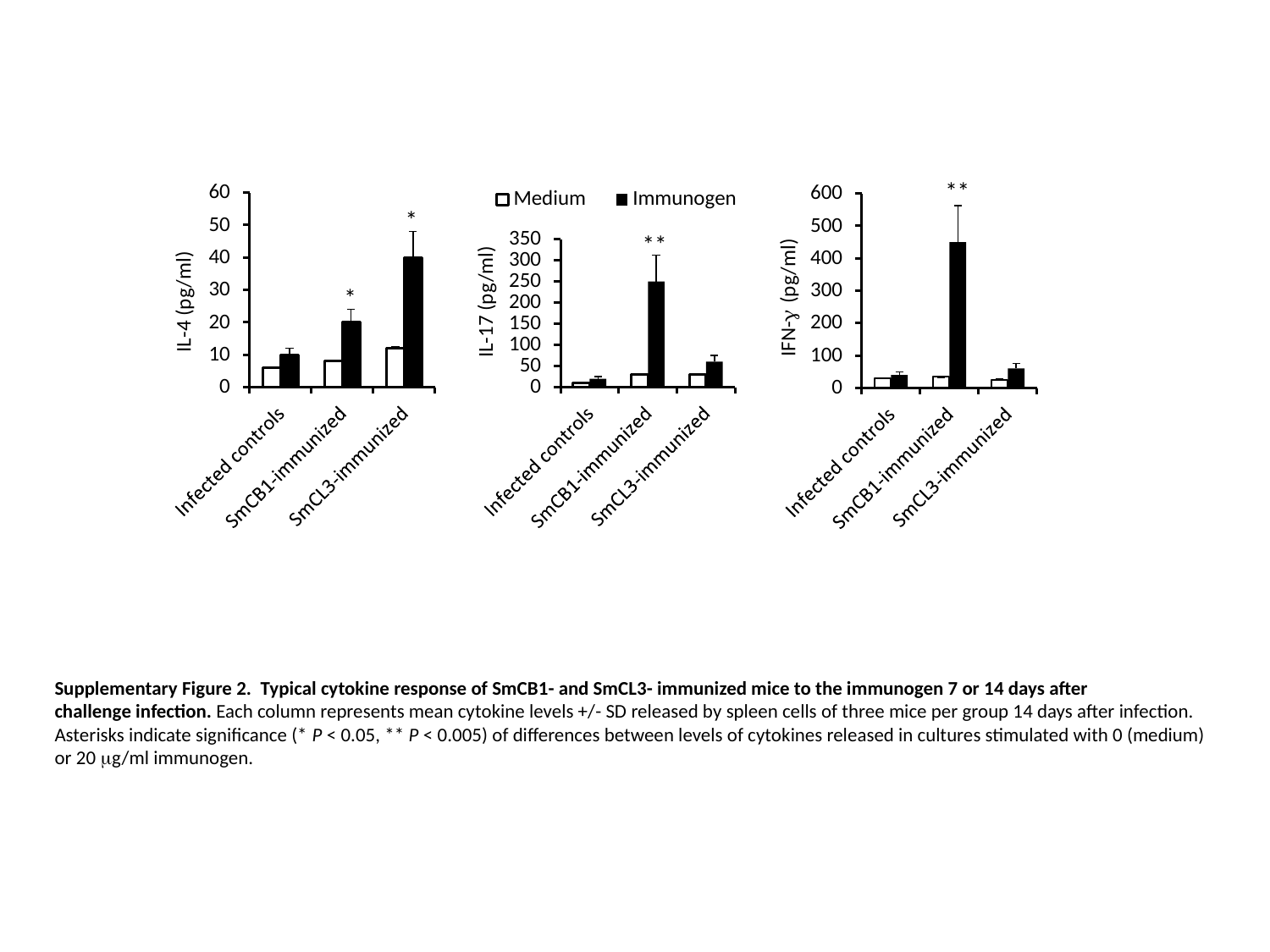

**
*
**
*
Supplementary Figure 2. Typical cytokine response of SmCB1- and SmCL3- immunized mice to the immunogen 7 or 14 days after
challenge infection. Each column represents mean cytokine levels +/- SD released by spleen cells of three mice per group 14 days after infection.
Asterisks indicate significance (* P < 0.05, ** P < 0.005) of differences between levels of cytokines released in cultures stimulated with 0 (medium)
or 20 mg/ml immunogen.
